# Unique genetic variants of lean nonalcoholic fatty liver disease: a retrospective cohort study

**DOI:** 10.1186/s12902-022-01234-w

**Published:** 2023-01-10

**Authors:** Jie Li, Na Wu, Yukun Yang, Xiangyu Zhai, Fan Yuan, Fengwei Zhang, Ning Yu, Dong Li, Ruirui Wang, Jianying Wang, Lei Zhang, Yi Shi, Guang He, Baocheng Liu

**Affiliations:** 1 Shanghai Innovation Center of Traditional Chinese Medicine Health Service, School of Public Health, Shanghai University of Traditional Chinese Medicine, Shanghai, China; 2 Bio-X Institutes, Key Laboratory for the Genetics of Developmental and Neuropsychiatric Disorders, Shanghai Jiao Tong University, Shanghai, China; 3 Changhai Hospital, Naval Military Medical University, Shanghai, China; 4 Graduate School of Sport Sciences, Waseda University, Saitama, Japan; 5 Zhangjiang Community Health Service Center of Pudong New District, Shanghai, China

**Keywords:** *FTO*, *GCKR*, Genetic risk, Lean NAFLD, Low-density lipoprotein

## Abstract

We investigated the prevalence and clinical metabolic characteristics of lean nonalcoholic fatty liver disease (NAFLD) in an elderly Chinese population and assessed the relevance of lipid markers and genetic variation. All 5,338 community subjects underwent detailed clinical and laboratory examinations and were divided into three groups: lean (Body mass index (BMI) < 23 kg/m^2^, *n* = 2,012), overweight (BMI = 23–24.9 kg/m^2^, *n* = 1,354), and obese (BMI ≥ 25 kg/m^2^, *n* = 1,972). Single nucleotide polymorphisms were selected based on those reported in previous NAFLD or obesity genome-wide association studies. The frequencies of alleles and genotypes were calculated and statistically analyzed with Pearson’s χ^2^ tests. One-way ANCOVA was used to test the association between positive SNPs and metabolic parameters in lean NAFLD individuals. Our results showed that the C allele frequency of rs2279026, the G allele of rs2279028, the C allele of rs780093, and the C allele frequency of rs1260326 were higher in obese NAFLD than in lean NAFLD (P < 0.05). In addition, we observed an association between the CC of rs1421085, TT of rs3751812, AA of rs8050136, and AA of rs9939609 genotypes in the *FTO* gene and low-density lipoprotein levels (*P* < 0.05). In conclusion, our findings provide a unique perspective on the prevalence, genetic characteristics, and metabolic profile of NAFLD in older lean individuals in China. This is the first study to examine the association between genetic variants in the *FTO*, *TFAP2B* and *GCKR* genes and NAFLD in a cohort of lean individuals.

**Supplementary Information:**

The online version contains supplementary material available at 10.1186/s12902-022-01234-w.

## Introduction

Nonalcoholic fatty liver disease (NAFLD) is one of the most widespread chronic liver diseases worldwide [1], the global prevalence of NAFLD is 25.24% [2]. Especially in China, the prevalence has reached 29.2% in 2019 [3]. NAFLD can range from simple steatosis to nonalcoholic steatohepatitis (NASH), which progresses to severely progressive liver cirrhosis and hepatocellular carcinoma [4]. NAFLD is strongly related to obesity, type 2 diabetes mellitus (T2DM), cardiovascular disease, dyslipidemia, and hypertension [5, 6]. Even though NAFLD often arises in the context of obesity, it also can be found in non-obese/lean subjects and presents with disease severity similar to that of obese NAFLD subjects [7]. Diagnosing NAFLD early is essential to halt the progression. Body mass index (BMI) is one of the etiological markers [8], however, it was found that using BMI to determine excess adiposity has limited sensitivity due to its inability to distinguish between fat and lean mass [9]. We attempted to explore a more precise and plausible metric that could be widely used for the diagnosis and assessment of disease for lean NAFLD.

Genetic variation contributes to differences in phenotypic traits in humans [10]. The pathogenesis underlying NAFLD progression and occurrence can be understood better by studying genetic factors that lead to NAFLD [11]. By interlocking and mutually reinforcing pathways, abnormal lipid metabolism, insulin resistance, and oxidative stress, along with inflammation, contribute to NAFLD [12]. As is well known, alterations in lipid metabolism are fundamental to the development and progression of NAFLD [13]. Hitherto, the best-known NAFLD inherited components are represented by single nucleotide polymorphisms (SNPs) in genes regulating hepatic lipid *accumulation* and metabolism, among which FTO alpha-ketoglutarate dependent dioxygenase (*FTO*) [14], transcription factor AP-2 beta (*TFAP2B*) [15], TBC1 domain family member 1 (*TBC1D1*) [16], glucokinase regulator (*GCKR*) [17], and potassium inwardly-rectifying channel subfamily J member 11 (*KCNJ11*) [18] have been evaluated as genetic determinants of NAFLD. In obese and lean individuals, nonsynonymous variants of the *FTO* gene are found identically, suggesting that the *FTO* protein itself might not be the sole explanation for the association of the *FTO* locus with obesity in humans [19, 20].

There is also the possibility of NAFLD occurring in lean individuals with genetic backgrounds, contributing to similar liver characteristics. The interaction of BMI and genetic factors in Asians warrants further investigation. We hope to discover the specific genetic variant of lean NAFLD by studying the metabolic and genetic variation characteristics of NAFLD so that we can better explain the pathogenesis of lean NAFLD. Therefore, we aimed to investigate the interaction of BMI with several known or potential genetic risk variables in a general population cohort regarding susceptibility and lipid metabolism in NAFLD and lean NAFLD. This study provides complete information on genetic polymorphism in the general Asian population and elucidates the genetic risk of NAFLD in lean individuals.

## Materials and methods

### Subjects

**Fig. 1:**
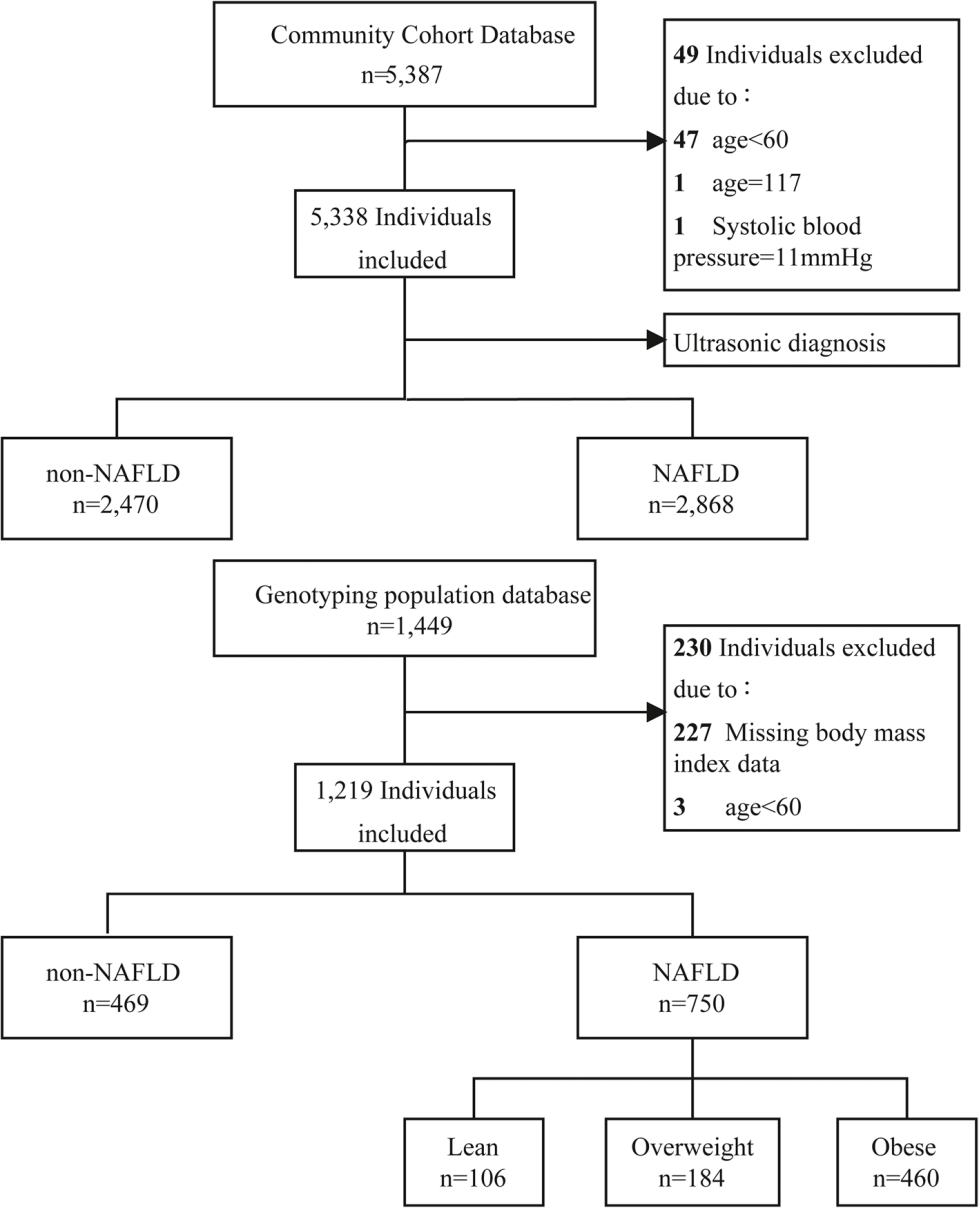
Data analysis flow chart. Abbreviations: NAFLD, Nonalcoholic fatty liver disease

This was a post-doc analysis of a cross-sectional population study conducted in Shanghai, China. We recruited 5387 residents from the Zhanjiang community of Shanghai aged ≥ 60 years and older between April and July 2017 for our study (Fig. 1). The study followed the Helsinki Declaration. A standard protocol has been developed by the Shanghai Innovation Center of Traditional Chinese Medicine Health Service and approved by the Ethics Committee of the Shanghai University of Traditional Chinese Medicine. All study participants provided informed consent. Participants with age ≥ 60 years, who live in Shanghai, can complete measurements and informed consent was included in the inclusion criteria. This study excluded participants with mental disorders, malignant tumors, or incomplete medical records. During the investigation, 49 subjects were excluded, resulting in a total of 5338 Chinese elderly subjects with complete data (2470 non-NAFLD and 2868 NAFLD).

In 2017, 1449 participants from the community cohort database who meet the following criteria were genotyped: permanent residents of Zhangjiang area of Pudong District, Shanghai; Chinese Han population without blood relations; and all medical examination, biochemical, and specimen collection information were complete and accurate. We excluded subjects who abused alcohol (< 140 g/week in males and < 70 g/week in females), had hepatitis B or C or had drug-induced or autoimmune liver disease history. In the genotype population cohort, 230 subjects were excluded, including missing BMI data and those < 60 years of age. The diagnosis of NAFLD was made based on 2010 guidelines from the Chinese Medical Association for its management [21].

### Clinical data collection

Two experienced radiologists evaluated fatty liver using a color ultrasound system [22]. Based on the criteria above, with NAFLD with a median (interquartile range) age of 69.02 (62.10–75.94) healthy controls from the same geographical area with a median age of 69.39 (62.03–76.75) were included in this study.

Questionnaires were used to collect information such as age, gender, alcohol consumption, current smoking, and medical history. We measured height and body weight using electronic measurement instruments (Sheng-yuan, Zhengzhou, China) wearing light clothing without hats or shoes, to the nearest 0.1 cm and 0.1 kg, respectively. Based on the BMI (body weight in kilograms divided by height in meters squared (kg/m2)), three categories were established: lean (BMI < 23 kg/m^2^), overweight (BMI between 23 and 24.9 kg/m^2^), and obese (BMI ≥ 25 kg/m^2^) [23] patients with NAFLD. Electronic sphygmomanometers were used to measure blood pressure (Bio-space, Cheonan, South Korea). We collected blood samples from the antecubital vein after fasting overnight in the morning. The following parameters were measured using an automatic biochemistry analyzer (Hitachi, Tokyo, Japan): fasting glucose, hemoglobin, platelets, ureophils, creatinine, uric acid, total cholesterol (TC), total bilirubin (TB), triglyceride (TG), low-density lipoprotein (LDL), high-density lipoprotein (HDL), alanine transaminase (ALT), and aspartate transaminase (AST).

### Extracting DNA, selecting SNPs, and genotyping

The genomic DNA was extracted from peripheral venous blood using the standard phenol–chloroform method for genotyping by collecting 5 mL of blood from each subject. The SNPs selected are based on positive reports of NAFLD or obesity from previous genome-wide association studies (GWAS) [14–20, 24, 25]. In this study, we examined the role of genes *FTO*, *TFAP2B*, *TBC1D1*, *GCKR*, and *KCNJ11* in NAFLD in Chinese Han individuals (Table 1). Twelve SNPs (rs1421085, rs3751812, rs8050136, and rs9939609 in the *FTO* gene, rs2206277 in the *TFAP2B* gene, rs2279027, rs2279026, and rs2279028 in the *TBC1D1* gene, rs780093, rs780094, and rs1260326 in the *GCKR* gene, and rs5215 in the *KCNJ11* gene) were genotyped in this study. With the Mass ARRAY® Analyzer 4 platform (Sequenom, San Diego, CA), all 12 of these SNPs have successfully been genotyped by matrix-assisted laser desorption/ionization-time of flight (MALDI-TOF) mass spectrometry. Probes and primers were designed using Mysequenom online software along with Assay Design Suite v2.0. Standard polymerase chain reaction (PCR) was carried out in a volume of 5 μL containing 10 ng of genomic DNA. The primers and conditions of the PCR reactions are available upon request.

**Table 1:** The 12 SNPs in the five genes evaluated in this cohort study

Gene	SNP ID^a^	Alleles	Chromosome^b^	Function
*FTO*	rs1421085	T/C	16:53,767,042	intron variant
*FTO*	rs3751812	G/T	16:53,784,548	intron variant
*FTO*	rs8050136	C/A	16:53,782,363	intron variant
*FTO*	rs9939609	T/A	16:53,786,615	intron variant
*TFAP2B*	rs2206277	C/T	6:50,830,813	intron variant
*TBC1D1*	rs2279027	T/A	4:37,902,135	missense variant
*TBC1D1*	rs2279026	T/C	4:37,902,461	synonymous variant
*TBC1D1*	rs2279028	A/C	4:37,902,048	genic upstream transcript variant
*GCKR*	rs780093	T/C	2:27,519,736	intron variant
*GCKR*	rs780094	T/C	2:27,518,370	intron variant
*GCKR*	rs1260326	T/C	2:27,508,073	missense variant
*KCNJ11*	rs5215	C/T	11:17,387,083	missense variant

^a^According to the dbSNP database

^b^The SNP Chromosome positions are based on the NCBI human genome

### Statistical analysis

All statistical tests were conducted using SPSS Statistics (IBM, version 26.0, Armonk, NY, USA). The continuous variables were expressed as mean ± standard deviation and compared using the Mann–Whitney U test or independent sample t-test as appropriate. Categorical variables were compared using the χ^2^ test. We tested the association between positive SNPs and metabolic parameters in lean NAFLD individuals using a one-way ANCOVA adjusted for age. Analyzing the distributions of alleles and genotypes was performed using SHEsis (http://analysis.bio-x.cn/my Analysis.php) (Bio-X Institutes, Shanghai, China) [26]. Categorical variables were expressed as counts or percentages and compared using Pearson’s χ^2^ tests. Hardy–Weinberg equilibrium (HWE) was assessed by Pearson’s χ2 tests. As well as odds ratios, 95% confidence intervals (CIs) were calculated. There was a statistical significance of *P* < 0.05.

## Results

### Demographics and clinical metabolic phenotypes

The overall clinical and laboratory characteristics of the 2470 non-NAFLD subjects and 2868 NAFLD subjects are listed in Table 2. The mean age of the subjects was 69.19 ± 7.13 years, and 2400 (45.0%) were male (Table 2). A total of 1324 (24.8%), 2861 (53.6%), 833(15.6%), 892 (16.7%), and 281 (5.3%) subjects had metabolic syndrome, hypertension, diabetes, cardiopathy, and hyperlipidemia, respectively. The subjects with NAFLD had a higher prevalence of metabolic syndrome, hypertension, and diabetes compared with the subjects without NAFLD (*P* < 0.001). A total of 2012 (37.7%) subjects had BMI < 23 kg/m^2^, 1354 (25.4%) subjects with BMI ranging from 23 to 24.9 kg/m^2^ and 1972 (36.9%) subjects with BMI ≥ 25.0 kg/m^2.^ The prevalence of NAFLD was 19.2% in lean subjects, 28.6% in overweight subjects, and 52.2% in obese subjects (*P* < 0.001). As shown in Table 2, the subjects with NAFLD tended to have significantly higher BMI, waist circumference (WC), waist-to-hip ratio, systolic blood pressure, and diastolic blood pressure compared with the subjects without NAFLD (*P* < 0.001). Patients with NAFLD were significantly taller and presented higher mean values of ALT, fasting glucose, hemoglobin, platelet, TC, LDL, TG, and uric acid, and lower mean values of creatinine and HDL than non-NAFLD.

**Table 2:** Medical history, demographic, and metabolic parameters of all individuals

Variables	Total	Non-NAFLD	NAFLD	*P* Value
N	5338	2470	2868	
***n (%)***				
Metabolic syndrome^a^	1324(24.8)	309(12.5)	1015(35.4)	< 0.001
Hypertension	2861(53.6)	1131(45.8)	1730(60.3)	< 0.001
Diabetes	833(15.6)	308(12.5)	525(18.3)	< 0.001
Cardiopathy	892(16.7)	432(17.5)	460(16.0)	0.157
Hyperlipidemia	281(5.3)	128(5.2)	153(5.3)	0.803
Male	2400(45.0)	1170(47.4)	1230(42.9)	0.001
Body mass index (kg/m^2^)				< 0.001
< 23 lean	2012(37.7)	1461(59.1)	551(19.2)	
23–24.9 overweight	1354(25.4)	535(21.7)	819(28.6)	
≥ 25 obese	1972(36.9)	474(19.2)	1498(52.2)	
***Mean* ± *SD***				
Age (years)	69.19 ± 7.13	69.39 ± 7.36	69.02 ± 6.92	0.062
Systolic blood pressure (mmHg)	142.14 ± 22.19	139.22 ± 22.81	144.66 ± 21.33	< 0.001
Diastolic blood pressure (mmHg)	81.51 ± 11.78	80.58 ± 12.08	82.32 ± 11.45	< 0.001
Waist circumference (cm)	82.64 ± 9.26	78.36 ± 8.52	86.34 ± 8.22	< 0.001
Waist-to-hip ratio	0.88 ± 0.07	0.86 ± 0.07	0.9 ± 0.06	< 0.001
ALT (U/L)	24.65 ± 21.72	22.34 ± 21.75	26.64 ± 21.49	< 0.001
AST (U/L)	23.88 ± 12.59	23.54 ± 12.31	24.17 ± 12.83	0.068
Ureophil (mmol/L)	5.48 ± 1.53	5.44 ± 1.56	5.51 ± 1.5	0.107
Fasting glucose (mmol/L)	6.16 ± 1.72	5.91 ± 1.57	6.38 ± 1.8	< 0.001
Hemoglobin (g/L)	140.09 ± 14.15	138.61 ± 14.59	141.36 ± 13.63	< 0.001
Platelet (10^9^/L)	197.14 ± 61.21	193.19 ± 67.7	200.55 ± 54.79	< 0.001
Total cholesterol (mmol/L)	5.08 ± 0.98	5.05 ± 1	5.11 ± 0.96	0.026
Total bilirubin (µmol/L)	15.78 ± 5.78	15.85 ± 5.77	15.72 ± 5.8	0.387
Creatinine (µmol/L)	69.8 ± 21.75	71.01 ± 25.5	68.77 ± 17.84	< 0.001
HDL (mmol/L)	1.25 ± 0.28	1.31 ± 0.29	1.19 ± 0.25	< 0.001
LDL (mmol/L)	3.17 ± 0.87	3.13 ± 0.88	3.21 ± 0.86	< 0.001
TG (mmol/L)	1.48 ± 1.09	1.21 ± 0.87	1.71 ± 1.2	< 0.001
Uric Acid (µmol/L)	344.36 ± 86.64	327.82 ± 82.36	358.61 ± 87.71	< 0.001

Continuous variables were expressed in mean ± standard deviation or median (interquartile range) and compared using the independent sample t-test as appropriate. Categorical variables were compared using the χ2 test

*Abbreviations: HDL* High-density lipoprotein, *LDL* Low-density lipoprotein, *TG* Triglycerides, *ALT* Alanine aminotransferase, *AST* Aspartate aminotransferase

*P*-value (NAFLD vs Non-NAFLD)

*P* < 0.05 was identified as a statistical significance

^a^International Diabetes Federation criteria for metabolic syndrome

### Genotyping of NAFLD risk loci

To determine if the genetic underpinning of NAFLD differs from the genetic underpinning of non-NAFLD subjects, we analyzed SNPs that have previously been identified as related to NAFLD and obesity in GWAS [12–18, 22, 23]. In the genotyped population cohort, there were eventually 750 subjects with NAFLD and 469 subjects with non-NAFLD included in the analysis. For the rs1421085 (73.9% for TT, 24.6% for CT and 1.3% for CC), rs3751812 (73.5% for GG, 25.1% for GT and 1.3% for TT), rs8050136 (74.1% for CC, 24.4% for CA and 1.3% for AA), rs9939609 (73.4% for TT, 1.3% for AA and 25.2% for AT), rs2206277 (35.8% for CT, 8.0% for TT and 56.1% for CC) and rs1260326 (18.6% for CC, 29.9% for TT and 51.4% for CT), all of these genotype groups showed significant prevalence in NAFLD subjects (*P* < 0.05) (See Supplementary file 1).

The allele frequencies of rs1421085, rs3751812, rs8050136, rs9939609, and rs2206277 were significantly different between NAFLD and non-NAFLD (*P* < 0.05). The C allele frequency of rs1421085 was remarkably higher in NAFLD individuals (OR = 1.407; 95%CI = 1.083–1.828; P = 0.010). The occurrence of T allele of rs3751812 was significantly increased in NAFLD group (OR = 1.443; 95% CI = 1.114–1.869; P = 0.005). In rs8050136, the A allele frequency was remarkably higher in NAFLD (OR = 1.430; 95% CI = 1.099–1.861; P = 0.007). And the A allele frequency of rs9939609 was also significantly increased in NAFLD (OR = 1.429; 95% CI = 1.105–1.849; *P* = 0.006). The T allele frequency of rs2206277 was remarkably higher in NAFLD individuals (OR = 1.305; 95%CI = 1.074–1.586; *P* = 0.007) (See Supplementary file 1).

### Genotyping of lean NAFLD risk loci

We separated the NAFLD population into three categories for subgroup analysis: lean NAFLD (*n* = 106), overweight NAFLD (n = 184), and obese NAFLD (*n* = 460). Importantly, the allele frequencies of rs2206277, rs2279026, rs2279028, rs780093, rs1260326, and rs5215 were different between overweight/ obese NAFLD and lean NAFLD (*P* < 0.05). The C allele frequency of rs2279026 (OR = 1.501; 95%CI = 1.074–2.098; *P* = 0.016), the G allele frequency of rs2279028 (OR = 1.516; 95%CI = 1.084–2.119; *P* = 0.014), the C allele frequency of rs780093 (OR = 1.381; 95%CI = 1.013–1.883; *P* = 0.040), and the C allele frequency of rs1260326 (OR = 1.376; 95%CI = 1.011–1.874; *P* = 0.041) were higher in obese NAFLD than lean subjects presenting NAFLD (See Supplementary file 2). When compared to lean NAFLD, the occurrence of C allele of rs780093 was significantly increased in overweight subjects presenting NAFLD (OR = 1.425; 95%CI = 1.006–2.019; P = 0.045) (See Supplementary file 2). Compared to lean NAFLD subjects, the T allele frequency of rs2206277 and the C allele frequency of rs5215 were both significantly lower in obese subjects presenting NAFLD (OR = 0.705, 95%CI = 0.521–0.953; *P* = 0.022; OR = 0.671; 95%CI = 0.484–0.931; *P* = 0.016) (See Supplementary file 2). There was no genotyping difference between lean NAFLD and overweight/obese NAFLD in the HWE paradigm.

### Association of genotypes and the phenotype in lean subjects

**Fig. 2:**
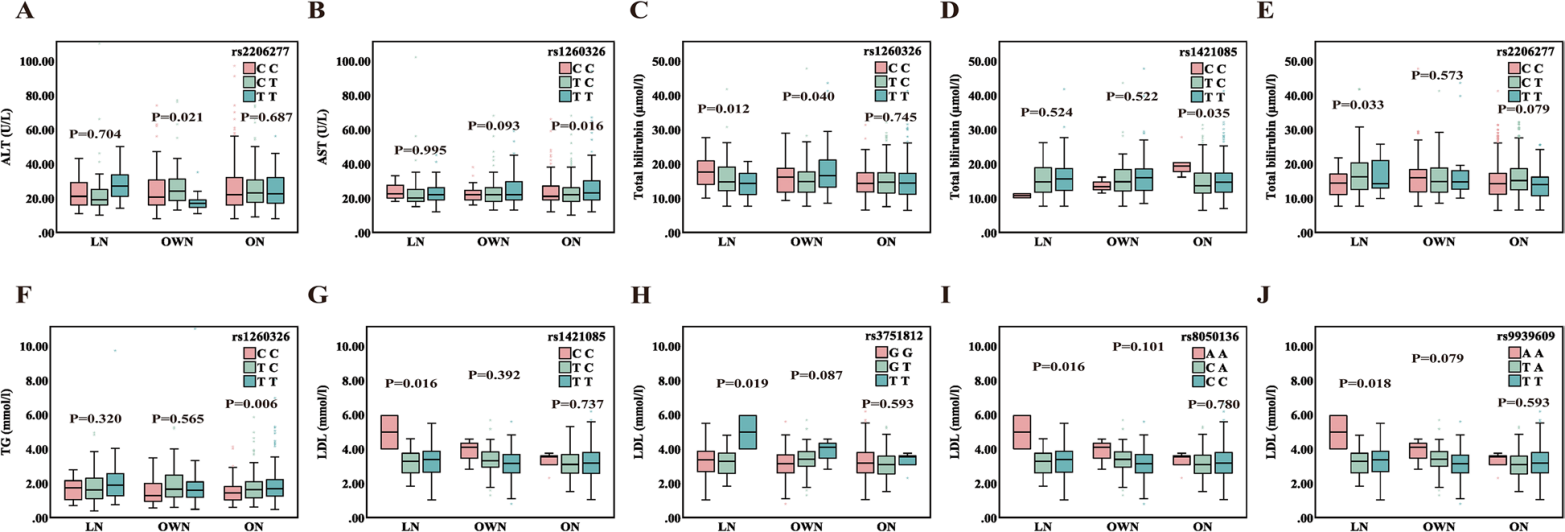
Association between clinical metabolic phenotypes with the *FTO*, *TFAP2B*, and *GCKR* genotype, stratified by BMI. Abbreviations: LN, Lean NAFLD; OWN, Overweight NAFLD; ON, Obese NAFLD; HDL, high-density lipoprotein; LDL, low-density lipoprotein; TG, triglycerides; ALT, alanine aminotransferase; AST, aspartate aminotransferase. *P*-value: Adjusted for age in ANCOVA. *P* < 0.05 was identified as a statistical significance

*TFAP2B* rs2206277 was associated with ALT in overweight subjects (*P* = 0.021), however, the difference was not significant between the lean and the obese (Fig. 2A). The association of *GCKR* rs1260326 genotype with AST and TG was significant only in obese subjects (Fig. 2B, F). Additionally, we observed that *GCKR* rs1260326 was associated with TB in both lean and overweight subjects (*P* < 0.05, Fig. 2C). *FTO* rs1421085 was associated with TB in obese subjects (*P* = 0.035, Fig. 2D). The relationship between *TFAP2B* rs2206277 genotype and TB was significant in the lean group (*P* = 0.033, Fig. 2E). The association between four SNPs, i.e., rs1421085, rs3751812, rs8050136, and rs9939609 in the *FTO* gene and LDL was significant in lean subjects but not in overweight and obese subjects (*P* < 0.05, Fig. 2G-J). There was no significant association between rs1421085, rs3751812, rs8050136, and rs9939609 and *TFAP2B* rs2206277 genotypes and TG. In addition, no significant association was found between the genotypes of six SNPs and TC/HDL (See Supplementary file 3). There was no significant association between the *FTO* rs1421085, rs3751812, rs8050136, and rs9939609, as well as the *TFAP2B* rs2206277 genotype and TG. In addition, no significant association between the genotypes of the six SNPs with TC/HDL was found (See Supplementary file 3).

## Discussion

In the present cross-sectional study, we found that the prevalence of metabolic syndrome, hypertension, and diabetes was significantly higher in NAFLD subjects than in non-NAFLD, and NAFLD subjects had a significantly higher BMI, WC, waist-to-hip ratio, blood pressure, hemoglobin, platelets, ALT, fasting glucose, TC, LDL, TG, and uric acid, and lower creatinine and HDL. These results are consistent with other cohorts’ studies [27]. It suggests that inflammatory and lipid-related metabolic features are more pronounced in older adults who have developed NAFLD and, more importantly, that clinicians should pay more attention to the potentially controversial role that metabolic-related comorbidities may have in NAFLD disease.

To investigate the genetic basis of NAFLD versus non-NAFLD subjects, we found that genotypes and alleles at the rs1421085, rs8050136, rs3751812, rs9939609 for *FTO*, rs2206277 for *TFAP2B*, and rs1260326 for *GCKR* loci were associated with increased risk of NAFLD. This result has been partially confirmed by the previous study from our group [28]. Several studies have reported that the *FTO* gene is critical in regulating body weight and fat mass, and plays a role in the energetic metabolism and regulation of the organism’s homeostasis [29]. In addition, we focused on the association of *TFAP2B* genotype with genetic susceptibility to NAFLD. The prevalence of NAFLD has been proven to be involved in obesity, T2DM, hyperlipidemia, and insulin resistance [30]. The metabolic syndrome is as well considered to be a risk factor for NAFLD [31]. Based on genome-wide association analysis data, genetic variants in *FTO* and *TFAP2B*, previously known as obesity susceptibility loci, impinge independently on the risk of metabolic syndrome [31]. Studies from Asia have revealed that genetic predisposition to the *FTO* and *TFAP2B* genes contributed to a sex-dependent pattern of obesity-related traits and remarkable accumulation of abdominal fat [32, 33]. The transcription factor *TFAP2B* is involved in regulating adipocyte metabolism by stimulating glucose uptake and lipid accumulation while decreasing insulin sensitivity [34]. Reduced expression of *TFAP2B* may have a protective effect against diminished insulin sensitivity and central obesity-related complications such as T2DM and coronary artery disease [34, 35]. Notably, a single mutant genetic variant rs1260326 in the glucokinase regulatory protein gene *GCKR* is implicated in the stress index of insulin resistance [36]. A retrospective cohort study from Iranian adult subjects identified a strongly associated *GCKR* variant with the prevalence of metabolic syndrome [37], and genetic variants of *GCKR* have been demonstrated to be involved in hepatic redox and contribute causally to key metabolic traits and diseases [38]. As hepatocyte lipid deposition is the initiating step and hallmark of NAFLD etiopathology [39], all three genes are involved in lipid metabolic pathways, such as adipogenesis, which are intimately associated with insulin resistance and NAFLD, all these facts directly or indirectly supported our finding.

To present, the relevance of SNPs to NAFLD has not been addressed in Chinese lean adults. In our contextual research sample, 19.2% of the NAFLD population suffered from lean NAFLD. Globally, about 40% of the global NAFLD population belongs to the non-obese population, and nearly one-fifth falls into the lean individuals [40]. Lean NAFLD subjects may have a more severe histological phenotype, higher mortality, and morbidity, and an increased risk of developing metabolic disease and progressing to more severe liver disease compared to overweight and obese NAFLD subjects [41, 42]. Several clinical, genetic, and metabolic findings suggest that NAFLD in lean individuals appears to be particularly warranted. In this study, we found that C allele rs2279026 of the *TBC1D1* gene, G allele rs2279028 of the *TBC1D1* gene, C allele rs780093 of the *GCKR* gene, and C allele rs1260326 of the *GCKR* gene were associated with a decreased risk of lean NAFLD in Chinese elderly population, T allele rs2206277 of the *TFAP2B* and C allele rs5215 of the *KCNJ11* were combined with an increased risk of lean NAFLD. Alternatively, in a subgroup analysis of the NAFLD population, we found that the C allele of rs2279026 of the *TBC1D1* gene, the G allele of rs2279028 of the *TBC1D1* gene, the C allele of rs780093 of the *GCKR* gene, and the C allele of rs1260326 of the *GCKR* gene were strongly associated with increased risk of NAFLD in obesity. The C allele of rs780093 of the *GCKR* gene was substantially more prevalent in overweight NAFLD. Genetic studies have shown that sterol regulatory protein binding factor (*SREBF*), transmembrane superfamily member 2 (*TM6SF2*), cholesteryl ester transfer protein, apolipoprotein C3 (*APOC3*), and patatin-like phospholipase domain-containing 3 (*PNPLA3*) have a promising contribution to the development and long-term prognosis of NAFLD in obese and lean individuals [43]. In a recently conducted investigation of risk variables for NAFLD in lean Chinese adults, investigators found no genotypic significant differences in common SNPs (sirtuin 1 (*SIRT1*) rs2273773, *APOC3* rs2070666, *PNPLA3* rs738409, *PNPLA3* rs738408, *PNPLA3* rs4823173, *PNPLA3* rs2072906, angiotensin II receptor type 1 (*AGTR1*) rs5186, and *AGTR1* rs440881) between lean subjects with and without NAFLD [44]. There is a similarity between such results and ours. Finally, our study complements the contribution of genetic factors at select loci in NAFLD in Chinese lean individuals, even due to the limited number of SNPs we measured.

Lean NAFLD subjects may have clinically similar metabolic dysfunction to obese or overweight individuals [45], even if they are at a much higher risk of developing metabolic dysfunction [46]. The pathogenesis of NAFLD in lean and obese individuals is not exclusively congruent. Regarding the majority of metabolic parameters, such as ALT, AST, TC, TB, TG, HDL, and LDL, there appeared to be discrepancies between the lean NAFLD subjects and the overweight/obese ones [47]. We observed for the first time that GCKR genotype rs1260326 for CC and TFAP2B genotype rs2206277 for CT were associated with TB in lean subjects, in which addition we found that GCKR genotype rs1260326 for TT was associated with TB in overweight recipients and FTO rs1421085 for CC was associated with TB in obese participants. We first observed that GCKR genotype rs1260326 for CC and TFAP2B genotype rs2206277 for CT were associated with TB in lean subjects, in addition we also found that GCKR genotype rs1260326 for TT was associated with TB in overweight subjects and FTO rs1421085 for CC was associated with TB in obese subjects. Studies on the correlation between genotype and TB are currently poorly reported. Some investigators have found that the UGT1A1 gene is a dominant genetic determinant of bilirubin concentration and that the UGT1A1*28 genotype is associated with an increased likelihood of hyperbilirubinemia and shows different features of association among different races [48]. We found that *FTO*, *TFAP2B*, and *GCKR* genotype variants interacted with certain metabolic profiles in lean NAFLD subjects. The *FTO* genotype of rs1421085 for CC, rs3751812 for TT, rs8050136 for AA, and rs9939609 for AA were associated with LDL levels, but no association was found between these genotypes and TG, TC, and HDL. The association of *FTO* genotype variants with lipid-related metabolic parameters has been documented, but mainly concerning overweight or obesity exposure, however, we must not discount that lean is also susceptible to lipid metabolic disorders [49], and we conducted the first analysis of the mutual interaction of *FTO* genotype variants with lean NAFLD and definite metabolic distinctions. A case–control analysis study of 612 Pakistani subjects found that the interaction between the CT genotype of rs1421085 and overweight/obesity affects TG and LDL independent of age and sex [50]. From another report, *FTO* gene expression was shown to be associated with LDL, and fasting glucose, but not TG, TC, and HDL [51]. We are also interested in an investigation of the association of the *FTO* rs9939609 polymorphism with lipid profiles in Iranian women, who noted that lower levels of HDL were observed in the AT/AA genotype compared to the TT wild-type genotype of the *FTO* rs9939609 polymorphism, with adjustments for age, BMI and physical activity that did not alter the results [52]. A cohort study of Polish Caucasians showed similar results, with GG genotype (rs3751812) and CC genotype (rs8050136) carriers having significantly higher TC and LDL levels when stratified into groups above the median fiber intake [53]. Given that our results revealed unique genetic factors and metabolic disorders in lean individuals with NAFLD, it would make sense to investigate this in Chinese subjects as well.

There are some limitations of this study. First, our diagnosis of NAFLD rested on ultrasound, and the assessment of the degree of visceral fat accumulation in the liver could not be ascertained. Despite invasive liver biopsy examinations may improve the precision and credibility of our data, but may not be appropriate for the large community cohort study. And ultrasonography is widely used in studies of large population cohorts and has some validity and feasibility. Second, insulin levels and insulin resistance were inadequately analyzed, even though diet and insulin resistance may be strongly associated with NAFLD in lean recipients. Notwithstanding the limitations, our findings provide critical insights into the morbidity, genetic characteristics, and metabolic facets of NAFLD in the Chinese elderly lean subjects. This is the first study to address the association of *FTO*, *TFAP2B*, and *GCKR* gene variants with NAFLD in a lean cohort.

## Conclusions

In conclusion, our findings provide unique perspectives on the morbidity, genetic characteristics, and metabolic profile of NAFLD in the Chinese elderly lean individuals. This is the first study to investigate the association of *FTO*, *TFAP2B*, and *GCKR* gene variants with NAFLD in a lean cohort. Notably, we observed an association between the CC of rs1421085, TT of rs3751812, AA of rs8050136, and AA of rs9939609 genotypes in the FTO gene and LDL levels. Future studies will be required to fully elucidate the role of gene-environment interactions in NAFLD and pathogenesis with a multi-omics approach.

## Supplementary Information

**Additional file 1: Supplementary file 1.** Genotype and allele frequencies of polymorphisms genetic variants in NAFLD subjects.

**Additional file 2:** **Supplementary file 2.** Genotype and allele frequencies of polymorphisms genetic variants in NAFLD subjects stratified by BMI.

**Additional file 3: Supplementary file 3. **Association between clinical metabolic phenotypes with the FTO, TFAP2B, and GCKR genotype, stratified by BMI. 

## Data Availability

The de-identified data set is available on request to the corresponding author.

## Abbreviations

*AGTR1*: Angiotensin II receptor type 1
ALT: Alanine aminotransferase
AST: Aspartate aminotransferase
*APOC3*: Apolipoprotein C3
BMI: Body mass index
CI: Confidence intervals
*FTO*: FTO alpha-ketoglutarate dependent dioxygenase
*GCKR*: Glucokinase regulator
GWAS: Genome-wide association studies
HDL: High-density lipoprotein
HWE: Hardy–Weinberg equilibrium
*KCNJ11*: Potassium inwardly-rectifying channel subfamily J member 11
LDL: Low-density lipoprotein
NAFLD: Nonalcoholic fatty liver disease
NASH: Nonalcoholic steatohepatitis
OR: Odds ratios
*PNPLA3*: Patatin-like phospholipase domain-containing 3
*SIRT1*: Sirtuin 1
SNPs: Single nucleotide polymorphisms
*SREBF*: Sterol regulatory protein binding factor
TB: Total bilirubin
TC: Total cholesterol
*TBC1D1*: TBC1 domain family member 1
T2DM: Type 2 diabetes mellitus
*TFAP2B*: Transcription factor AP-2 beta
TG: Triglycerides
*TM6SF2*: Transmembrane superfamily member 2
WC: Waist circumference
