# Supplementary material for: Unique genetic variants of lean nonalcoholic fatty liver disease: a retrospective cohort study
Source: BMC Endocr Disord. 2023 Jan 10;23:11. doi: 10.1186/s12902-022-01234-w (PMC9830772; doi:10.1186/s12902-022-01234-w)
Supplement: Supplementary file 3 — Additional file 3: Supplementary file 3. Association between clinical metabolic phenotypes with the FTO, TFAP2B, and GCKR genotype, stratified by BMI. [file 12902_2022_1234_MOESM3_ESM.pdf]

Supplementary File 3. Association between clinical metabolic phenotypes with the FTO, TFAP2B, and GCKR genotype, stratified by BMI.

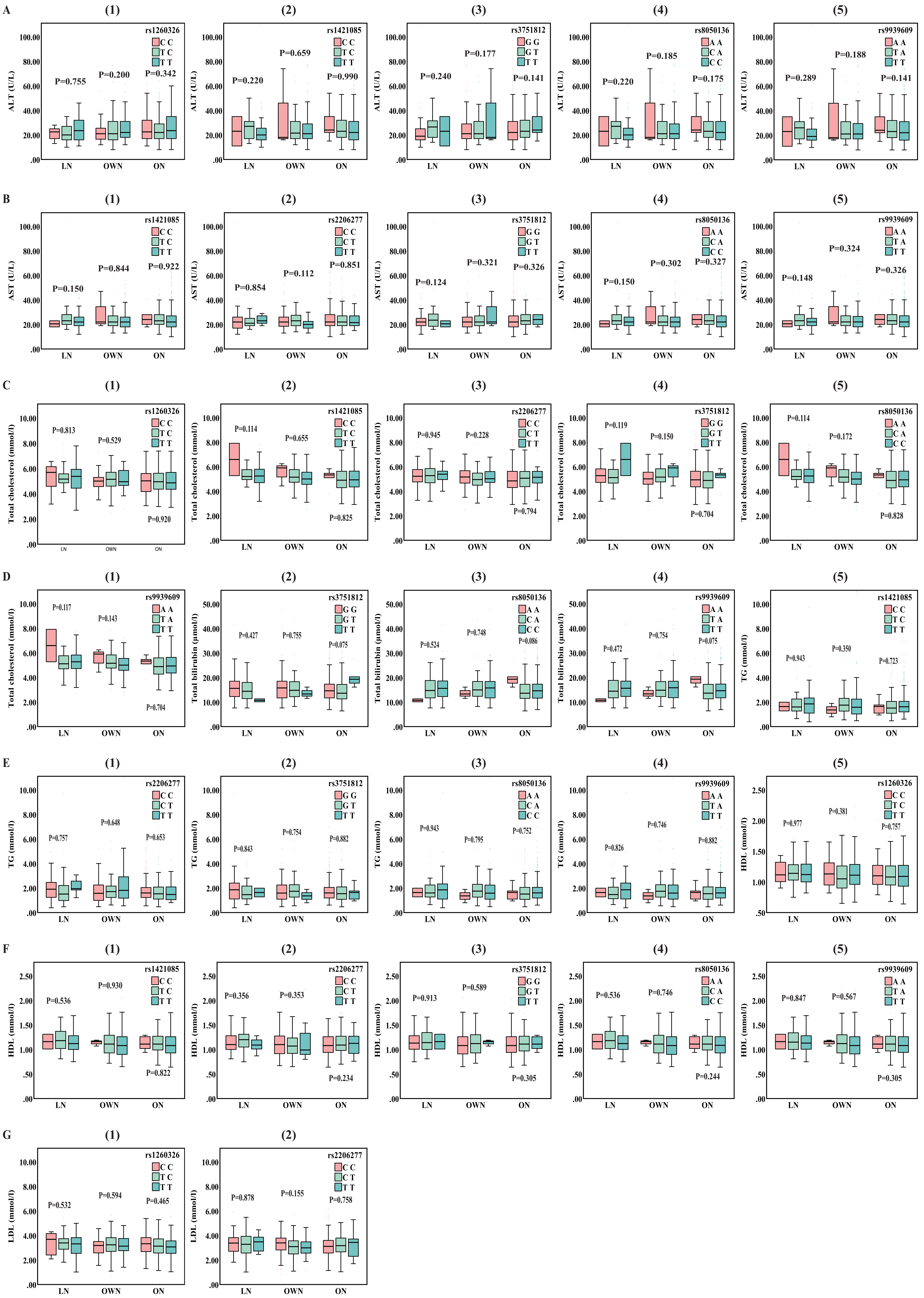

Abbreviations: HDL, high-density lipoprotein; LDL, low-density lipoprotein; TG, triglycerides; ALT, alanine aminotransferase; AST, aspartate aminotransferase.
P-value: Adjusted for age in ANCOVA. P < 0.05 was identified as a statistical significance.
